# A core outcome set for studies evaluating interventions to prevent and/or treat delirium for adults requiring an acute care hospital admission: an international key stakeholder informed consensus study

**DOI:** 10.1186/s12916-021-02015-3

**Published:** 2021-06-18

**Authors:** Louise Rose, Lisa Burry, Meera Agar, Bronagh Blackwood, Noll L. Campbell, Mike Clarke, John W. Devlin, Jacques Lee, John C. Marshall, Dale M. Needham, Najma Siddiqi, Valerie Page

**Affiliations:** 1 Florence Nightingale Faculty of Nursing, Midwifery and Palliative Care, King’s College London, Rm 1.13, James Clerk Maxwell Building, 57 Waterloo Rd, London, SE1 8WA UK; 2 Leslie Dan Faculty of Pharmacy, University of Toronto, Toronto, Canada; 3 Mount Sinai Hospital, Sinai Health System, Toronto, Canada; 4 Faculty of Health, University of Technology Sydney, Sydney, Australia; 5 Wellcome-Wolfson Institute of Experimental Medicine, Queen’s University Belfast, Belfast, Northern Ireland, UK; 6 College of Pharmacy, Purdue University, West Lafayette, IN USA; 7 School of Medicine, Dentistry and Biomedical Sciences, Queen’s University Belfast, Belfast, Northern Ireland; 8 School of Pharmacy, Northeastern University, Boston, MA USA; 9 Inaugural Research Chair in Geriatric Emergency Medicine, Schwartz/Reisman Emergency Medicine Institute, Sinai Health System, Toronto, Ontario Canada; 10 St Michael’s Hospital and Li Ka Shing Research Institute, Toronto, Canada; 11 School of Medicine, Johns Hopkins University, Baltimore, MD USA; 12 Hull York Medical School, University of York, York, UK; 13 Watford General Hospital, Watford, UK

**Keywords:** Delirium, Hospitalization, Core outcome set, Clinical trials

## Abstract

**Background:**

Trials of interventions to prevent or treat delirium in adults in an acute hospital setting report heterogeneous outcomes. Our objective was to develop international consensus among key stakeholders for a core outcome set (COS) for future trials of interventions to prevent and/or treat delirium in adults with an acute care hospital admission and not admitted to an intensive care unit.

**Methods:**

A rigorous COS development process was used including a systematic review, qualitative interviews, modified Delphi consensus process, and in-person consensus using nominal group technique (registration http://www.comet- initiative.org/studies/details/796).

Participants in qualitative interviews were delirium survivors or family members. Participants in consensus methods comprised international representatives from three stakeholder groups: researchers, clinicians, and delirium survivors and family members.

**Results:**

Item generation identified 8 delirium-specific outcomes and 71 other outcomes from 183 studies, and 30 outcomes from 18 qualitative interviews, including 2 that were not extracted from the systematic review. De-duplication of outcomes and formal consensus processes involving 110 experts including researchers (N = 32), clinicians (N = 63), and delirium survivors and family members (N = 15) resulted in a COS comprising 6 outcomes: delirium occurrence and reoccurrence, delirium severity, delirium duration, cognition, emotional distress, and health-related quality of life. Study limitations included exclusion of non-English studies and stakeholders and small representation of delirium survivors/family at the in-person consensus meeting.

**Conclusions:**

This COS, endorsed by the American and Australian Delirium Societies and European Delirium Association, is recommended for future clinical trials evaluating delirium prevention or treatment interventions in adults presenting to an acute care hospital and not admitted to an intensive care unit.

**Supplementary Information:**

The online version contains supplementary material available at 10.1186/s12916-021-02015-3.

## Background

Adults requiring admission to an acute care hospital who are at risk of delirium comprise a heterogeneous group including those undergoing major surgery [1], older adults with non-surgical indications for acute hospital admission such as pneumonia and urosepsis [2], and in recent times the SARS-CoV-2 virus [3]. Delirium is a syndrome characterized by fluctuating mental status with marked inattention and other cognitive disturbance [4] that is attributable to one or more etiologies. Post-operative delirium is a common complication, with prevalence as high as 50% depending on surgery type and patient risk [2]. Among older adults admitted to an acute care hospital ward, 1 in 5 experience delirium [5].

The consequences of delirium are serious and include neurocognitive disturbance and cognitive decline [6], prolonged hospitalization [7], discharge to post-acute care facilities, increased caregiver burden [8], decreased functional status [9, 10], adverse events such as falls [11], and mortality [12, 13]. As delirium persists, the risk of mortality at 6 months increases [14]. Many patients whose hospitalization is complicated by delirium never return to baseline functional status. Delirium poses substantial additional costs to healthcare systems; with US healthcare costs attributable to delirium estimated to exceed $182 billion annually [15, 16].

While strong evidence indicates delirium is partially preventable through multi-component nonpharmacologic approaches [17], pharmacological prevention or treatment strategies have yet to be proven effective [18, 19]. Disparate outcome selection in trials evaluating the same intervention is an important barrier to effectively synthesizing study results, precluding the ability to developing evidence-based practices and policies [20, 21]. Core outcomes sets (COS) are an agreed-upon minimum set of outcomes to be measured and reported in *all* studies relating to a specific health condition [22]. COS offer a solution to reducing heterogeneity of trial outcome selection. Therefore, our objective was to undertake a rigorous international consensus process for a COS for trials of interventions, designed to prevent and/or treat delirium, for adults requiring an acute care hospital admission, but who do not require intensive care unit (ICU) admission. We elected to develop a single COS for prevention and treatment trials as many evaluate an intervention as a continuum of prevention to treatment, particularly in participant groups (those receiving anesthesia or sedation, or those with concomitant cognitive issues) in whom early confirmation of delirium is challenging. We excluded trials conducted in the ICU in this COS as we hypothesized outcomes specific to critically ill patients such as ventilation duration might be considered important.

## Methods

We followed Core Outcome Measures in Effectiveness Trials (COMET) guidelines [23] for this COS development study and report on it in accordance with Core Outcome Set–STAndards for Reporting [24]. To commence the item generation process required for a COS, we conducted a systematic review of outcomes reported in published trials (1980 to December 2016) and registered trial protocols (January 2014 to December 2016) via (1) search term development in collaboration with two senior information specialists and conduct of the search across ten publication databases and grey literature; (2) two authors independently screening citations and extracting data on study characteristics, outcomes, and measures (with a third author as arbiter if needed); and (3) assignment of outcomes according to COMET taxonomy [25]. We included randomized, quasi-randomized, or non-randomized intervention studies of pharmacological (e.g., haloperidol) or non-pharmacological (e.g., reorientation, music) interventions for delirium prevention, treatment, or both, conducted in adults or children experiencing an acute hospital admission. We excluded studies conducted in ICUs and those reporting interventions to treat pediatric or adult agitation on emergence from general anesthesia. In addition, our item generation process included semi-structured qualitative interviews exploring outcomes important to delirium survivors and family members.

Item reduction and consensus methods comprised a two-round, web-based modified Delphi consensus process. To gain final consensus, this Delphi process was followed by an in-person consensus meeting, hosted by the European Delirium Association, using a modified nominal group technique [26].

### Recruitment of participants for qualitative interviews, Delphi panel, and consensus meeting

We sought a purposive and international sample from three stakeholder groups: (1) clinical researchers, (2) clinicians, and (3) delirium survivors and family. We recruited delirium survivor and family participants using a multi-modal strategy, including a designated study Twitter account, snowballing (i.e., research participants passing on recruitment materials to other potential participants), and personal contacts. Our multi-modal strategy to recruit expert clinicians and delirium researchers included recruitment flyers sent through membership lists of the American Delirium Society and Australian Delirium Association and to attendees of the European Delirium Association 2019 meeting (in-person consensus meeting), announcements at the American Delirium Society 2019 meeting, personalized recruitment emails sent to corresponding authors of studies included in our systematic review, flyers posted in UK National Health Service organizations, snowballing, and personal contacts.

### Semi-structured interviews

Semi-structured interviews with delirium survivors and family members were conducted by telephone by a single experienced interviewer (LR). The interview guide incorporated COMET plain language [27] to orient participants to the terms “study outcomes” and “COS.” Interviews were audio recorded, transcribed verbatim, and content analyzed by one author [28].

### Delphi methods

Item reduction for identified outcomes occurred via de-duplication (i.e., removing redundant outcomes), removing outcomes related to aggregate population data rather than individual patient outcomes (e.g., number of patients receiving analgesia), and grouping similar outcomes [29]. We grouped into a single outcome those describing adverse events, side effects, and complications, and those describing study-related feasibility or process outcomes. As more items are associated with lower COS Delphi response rates [30], we further reduced outcomes by removing those identified in < 5% of studies, unless specifically mentioned in survivor/family member interview transcripts. The final list of outcomes was then reviewed for wording clarity (with lay descriptions of medical terms to aid understanding) and for domain grouping.

To conduct the Delphi, we used the bespoke DelphiManager software, Version 4 (COMET Initiative, Liverpool, UK). Participants were directed to self-select their key stakeholder group (i.e., patient/family; clinician; researcher) and to score the importance of each outcome for COS inclusion, without consideration of measurability or feasibility. Importance was scored using the Grading of Recommendations Assessment, Development and Evaluations (GRADE) Scale [31]. This is a 9-point Likert scale with scores 1 to 3 considered not important, 4 to 6 important but not critical, and 7 to 9 as critical for inclusion. This scoring method is recommended by COMET to facilitate maximum discrimination between questionnaire items [32, 33]. Participants were provided an “Unable to Score” response option and the opportunity to suggest additional outcomes. To avoid presentation bias, the DelphiManager software randomized outcome domain presentation order.

For Delphi round 1 scores, we calculated mean and standard deviation (SD) of GRADE importance scores and determined the proportion of participants rating each outcome with scores of 7 to 9 (critically important), 4 to 6 (important but not critical), and 1 to 3 (not important) for the entire expert panel, and separately for each stakeholder group. Additional suggested outcomes were deduplicated and worded appropriately for inclusion in round 2. For round 2, participants received their own round 1 scores and summarized scores, with visual representation using histograms. Participants were asked to re-score outcome importance. If a participant changed their scoring so that it moved into a new category (e.g., from “important but not critical” to “critical for inclusion”), participants were requested to provide a free-text reason for this change. For both rounds, we sent three email reminders regarding completion using the DelphiManager software.

### In-person consensus meeting and nominal group technique

To inform our in-person consensus meeting, we calculated mean (SD) Delphi round 2 importance scores and determined the proportion of participants rating each outcome as critical for inclusion overall and by stakeholder group. As recommended by COMET [22], outcomes brought to the consensus meeting met the following criteria: scored as “critical for inclusion” by ≥ 70% of respondents and “not important” by < 15% considering all participants and for each of the three key stakeholder groups. No outcome that was rated by < 70% of participants as critically important overall or within a stakeholder group was brought forward to the consensus meeting.

For pragmatic reasons, we timed our consensus meeting with the 2019 European Delirium Association annual conference. We provided an overview of our meeting’s aim and structure and the Delphi results. We provided the importance scoring for the outcomes by stakeholder group to consensus meeting participants, for consideration during outcome ranking. Using nominal group technique methods, we held iterative rounds of small group and then whole group discussion. To avoid negating the Delphi process, participants were not permitted to suggest new outcomes. Participants ranked outcomes from most critical to least critical for COS inclusion at the end of each discussion.

The study was funded by the Canadian Institutes of Health Research. It received approval from the Research Ethics Boards of the University of Toronto, King’s College London, Sunnybrook Health Sciences Centre (Toronto, Canada), and the UK Health Research Authority (HRA) and Health and Care Research Wales (HCRW). Ethics approvals to recruit via social media, snowballing, and networking methods enabled recruitment from multiple countries including the USA, Europe, Asia, Oceania, and South America. Written informed consent was obtained from all study participants. The Del-CORs project is registered with the COMET initiative (http://www.comet- initiative. org/studies/details/796). We previously published the study protocol [34].

## Results

### Item generation via systematic review

**Fig. 1:**
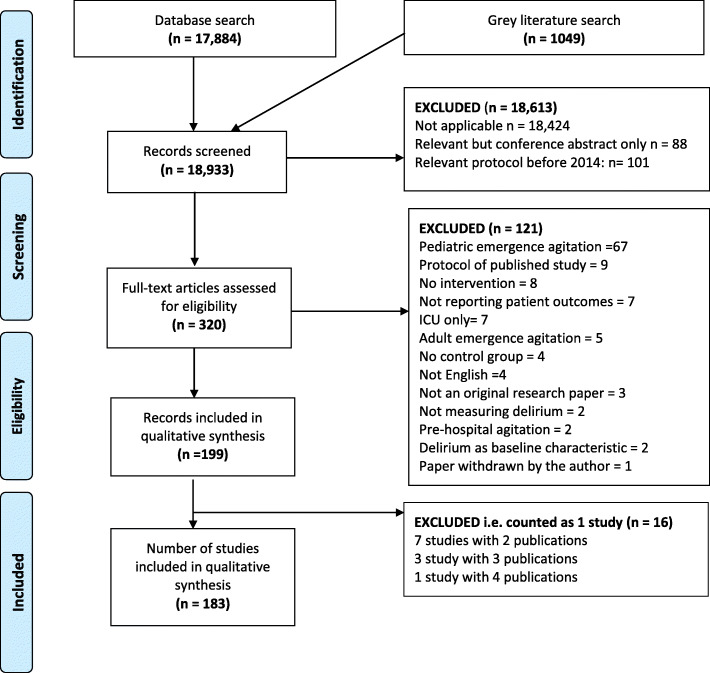
PRISMA flow diagram

We screened 18,933 citations, identified and extracted data pertaining to study outcomes and measures from 183 studies meeting our inclusion criteria (Fig. 1). Of the 183 included studies, 150 (88%) recruited older adults only (classified according to the study author’s participant description); most (109/183, 60%) were in post-operative patients. Delirium prevention was the primary intervention aim for 125 (68%) studies (Table 1). We extracted information on 79 outcomes reported in more than one study. These included 8 delirium-specific outcomes (Additional File Table 1) and 71 other outcomes categorized using COMET taxonomy [25] (Additional File Table 2).

**Table 1:** Systematic review study characteristics

*N* = 183 studies	n (%)
Study design	
RCT	128 (70)
Before and after intervention study	29 (16)
Non-randomized controlled trial	19 (10)
Other^a^	7 (4)
Study region	
North America	58 (32)
Europe	52 (28)
Asia	50 (27)
Oceania	15 (8)
Euroasia	5 (3)
South America	2 (1)
Multiple	1 (1)
Study population	
Adults only	183 (100)
Older adults only	150 (88)
Patient type	
Surgical	109 (60)
Medical	68 (37)
Both	6 (3)
Delirium as a study objective	
Primary	137 (75)
Secondary	46 (25)
Study intervention aim	
Prevention only	125 (68)
Treatment only	18 (10)
Both	44 (22)
Study intervention	
Pharmacological agent to prevent and/or treat delirium	87 (48)
Protocol or bundle to prevent and/or treat delirium	80 (44)
Non-pharmacological to prevent and/or treat delirium	16 (9)

^a^Other comprised: 5 interventional cohort studies with controls, 1 quasi RCT, and 1 interrupted time series study

Percentages may not sum to 100 due to rounding

### Item generation via interviews with delirium survivors and family members

We recruited 18 delirium survivors or family members from Canada, USA, and UK. From these interviews, 30 potential outcomes were identified (Table 2). The most commonly identified outcomes in the interview dataset were (1) “emotional distress, i.e., fear and anxiety related to delirium symptoms such as delusions” and (2) “delirium severity” (both identified by 50% of interview participants). Only two outcomes, “ability to return to previous lifestyle/work” and “subsyndromal delirium” (named as such by a family member with medical background) were not identified in the systematic review and brought forward for consideration for inclusion in the round 1 Delphi.

**Table 2:** Outcomes identified by delirium survivor and family interview participants

Outcome (*N* = 18 interview participants)	n (%)
Emotional distress (i.e., fear and anxiety related to delirium symptoms)	9 (50)
Delirium severity (i.e., severity of hallucinations, paranoid thoughts, delusions, disorientation)	9 (50)
Ability to get back to previous cognitive abilities/long term cognitive outcomes	8 (44)
Agitation—occurrence and duration^a^	8 (44)
Safety—falls and other injuries, pulling out lines	8 (44)
Ability to live alone independently/manage activities of daily living	7 (39)
Being able to mobilize/physical functioning after discharge	7 (39)
Risk factors for delirium including environmental factors^b^	6 (33)
Delirium duration	5 (28)
Repeated infection as a risk factor for delirium^b^	5 (28)
Delirium reoccurrence and its risk factors^b^	5 (28)
Sleep quantity and quality	5 (28)
Quality of life/recovery (physical and psychological)	5 (28)
Acute stress and post-traumatic stress disorder	4 (22)
Impact of delirium on family (stress, emotional wellbeing, burden)^a^	4 (22)
Length of stay	3 (17)
Mortality/survival	3 (17)
Use of chemical restraint/psychotropic drugs	3 (17)
Pain	3 (17)
Discharge disposition including ability to be discharged home	3 (17)
Time to/frequency of mobilization	3 (17)
Use of physical restraint	2 (11)
Ability to return to previous lifestyle/work^c^	2 (11)
Time to delirium diagnosis	2 (11)
Depression	1 (6)
Sedative dose	1 (6)
Delirium incidence	1 (6)
Delirium resolution	1 (6)
Subsyndromal delirium^c^	1 (6)
Hospital readmission	1 (6)

^a^Identified in systematic review but reported in < 5% of studies

^b^Considered as not an outcome during adjudication processes

^c^Not identified in systematic review

### Consensus building

Deduplication decisions (Additional File Table 3) resulted in selection of 31 outcomes for the Delphi round 1 (see outcomes listed in Additional File Table 4). We recruited 110 participants for the Delphi international expert panel; 15 (14%) were delirium survivors or family members of whom 7 were also healthcare providers or researchers (Table 3). Of the 31 outcomes provided in round 1, 20 (65%) met a priori consensus criteria for COS inclusion considering all participant responses, 8 (26%) by all three stakeholder groups (Additional file 1: Table 4). Compared to clinicians or survivors/family, researchers were less likely to consider outcomes critical for inclusion that related to emotional well-being, sleep, or agitation and its management (i.e., physical restraint).

**Table 3:** Round 1 Delphi participants

(*N* = 110)	n (%)
Country of residence	
USA	41 (37)
UK	21 (19)
Australia and New Zealand	20 (18)
Europe	11 (10)
Canada	11 (10)
South America	3 (3)
Asia/Middle East	3 (3)
Involvement with delirium	
Research and clinical work	73 (66)
Clinical work only	17 (15)
Delirium survivors and family members	15 (14)
Research work only	5 (5)
Profession of healthcare profession participants (N = 90)	
Physician	66 (73)
Nurse or nurse practitioner	14 (16)
Other healthcare profession	6 (7)
Physio, respiratory, or occupational therapist	4 (4)
Years of clinical experience (N = 90)	
> 10	73 (81)
6–10	13 (14)
3–5	4 (4)

For Delphi round 2, 8 additional outcomes were included based on suggestions in round 1. These included (1) development of incontinence, (2) nutritional status, (3) workload, (4) use of sitters, (5) family caregiver burden, (6) staff satisfaction, (7) new onset dementia, and (8) ability to participate in rehabilitation. Of the 110 round 1 participants, 77 (70%) participated in round 2. Of the 39 outcomes provided, 22 (56%) met consensus criteria for inclusion in the COS considering all participant responses, 17 (44%) by all three stakeholder groups (Table 4). Of the 8 added outcomes, none met inclusion criteria.

**Table 4:** Round 2 Delphi scores

Outcomes	Overall		Survivor/family (N = 7)	Clinician (N = 42)	Researcher (N = 28)
	Mean (SD)	% critical	% critical	% critical	% critical
Delirium occurrence	8.6 (0.8)	97	86	98	100
Delirium duration	8.0 (1.0)	95	86	95	96
Adverse events/side effects	8.0 (1.0)	95	86	97	93
Mortality	8.3 (1.0)	93	100	90	96
Cognitive status	8.1 (1.0)	92	100	88	96
Delirium severity	8.0 (1.0)	92	100	93	89
Delirium resolution	7.7 (1.1)	87	71	93	82
Agitation	7.5 (1.2)	87	100	92	75
Use of antipsychotics/other medication for agitation	7.6 (1.3)	84	86	88	79
Activities of daily living	7.7 (1.2)	83	71	88	86
Patient emotional wellbeing	7.5 (1.1)	82	100	85	71
Sleep	7.4 (1.3)	81	100	85	70
Hospital disposition	7.6 (1.3)	80	71	80	71
Falls and other injuries	7.6 (1.4)	80	100	80	75
Physical restraint	7.4 (1.6)	79	86	88	64
Physical functioning	7.3 (1.2)	78	71	78	79
Delirium reoccurrence	7.4 (1.3)	78	86	83	68
Length of stay	7.4 (1.3)	78	86	81	71
Hospital readmission	7.3 (1.4)	75	71	76	75
Health-related quality of life	7.2 (1.2)	75	100	83	57
Sedation score/level indicating quality of sedation	7.1 (1.5)	73	86	74	67
New onset dementia	7.0 (1.6)	71	100	64	71
Costs	7.0 (1.4)	64	57	67	61
Caregiver burden	6.8 (1.4)	61	71	66	50
Pain score/level indicating quality of analgesia	6.8 (1.5)	58	86	60	48
Pressure ulcers	6.7 (1.3)	52	71	53	46
Delirium type	6.5 (1.5)	50	86	44	50
Analgesic drug use	6.6 (1.5)	49	71	50	41
Ability to participate in rehab	6.5 (1.7)	48	71	46	46
Pneumonia	6.7 (1.4)	45	71	43	43
Time to delirium onset	6.3 (1.6)	45	86	54	21
Patient/family satisfaction	6.3 (1.5)	40	86	49	14
Workload	6.1 (1.6)	40	83	41	25
Study intervention related process outcomes	6.2 (1.4)	39	29	37	36
Use of sitters	6.0 (1.6)	39	71	36	30
Family emotional wellbeing	6.2 (1.3)	36	71	49	7
Nutritional status	6.0 (1.6)	36	71	32	32
Staff satisfaction	5.7 (1.7)	32	71	26	25
Incontinence	5.8 (1.4)	31	43	33	25

**Fig. 2:**
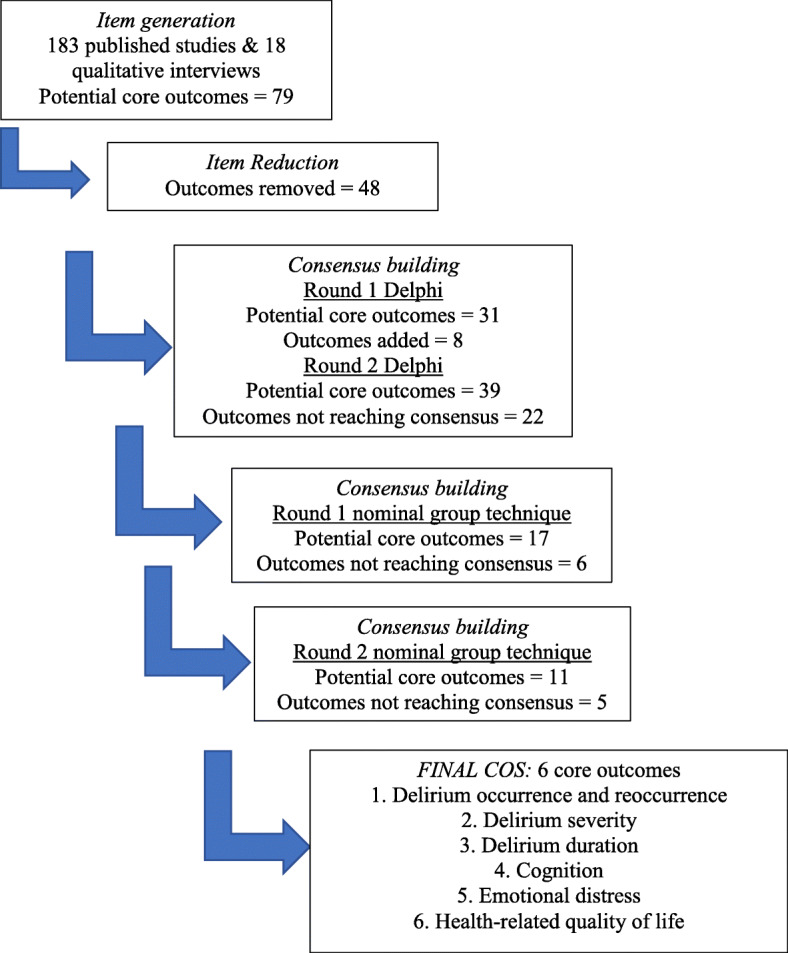
Development of COS for trials of interventions to prevent or treat delirium in hospitalized adults

Twelve experts (including 1 delirium survivor) participated in the in-person consensus meeting. After the first round of small and then large group discussion using nominal group technique ranking exercises, 6 of the 17 (35%) outcomes were excluded. “Falls and other injuries” and “agitation” were voted out by one small group but not the second. On further discussion and ranking, these outcomes as well as mortality (causes of falls and mortality were considered multi-factorial and not delirium specific) were excluded. Delirium occurrence and reoccurrence was collapsed into a single outcome. The 6 outcomes selected for the COS for trials of interventions to prevent and/or treat delirium in adults requiring an acute care hospital admission comprised: (1) delirium occurrence and reoccurrence, (2) delirium severity, (3) delirium duration, (4) cognition, (5) emotional distress, and (6) health-related quality of life (Fig. 2).

## Discussion

This study is the first to establish a COS for use in future efficacy and effectiveness trials of interventions focused on preventing and/or treating delirium experienced by adults required an acute care hospital admission, without an ICU admission. This study rigorously followed established COMET methods for COS development. We used systematic review methods and qualitative interviews with delirium survivors and family members to generate a comprehensive list of potential outcomes. Robust consensus building (Delphi and nominal group technique) methods were then used to establish six outcomes for inclusion in the final COS. This COS addresses one of the priority areas identified by the 2019 Scientific Think Tank report from NIH-funded Network for Investigation of Delirium: Unifying Scientists (NIDUS) group [35] and an Agency for Healthcare Research and Quality report recommendation [36]. The use of this COS in future trials of hospitalized adults is endorsed by the American and Australian Delirium Societies and European Delirium Association.

This COS includes three outcomes that characterize delirium itself, i.e., “occurrence and reoccurrence,” “severity,” and “duration.” Measurement of delirium occurrence, an umbrella term for incidence and prevalence, is imperative to determine the effect of interventions designed to prevent delirium. Delirium reoccurrence is relevant to trials of interventions designed to treat delirium. Fear of delirium reoccurrence was emphasized in interviews with delirium survivors and family members further highlighting the emotional distress associated with delirium. Inclusion of delirium severity reflects growing interest in its measurement as a way of understanding symptom burden, risk stratification, selection of appropriate interventions, clinical course, and prognosis [37]. Greater delirium severity is associated with worse outcomes, such as discharge to a post-acute healthcare facility and death. Similarly prolonged delirium duration is associated with worse outcomes after hospital discharge [38].

Inclusion of “cognition” and “health-related quality of life” as core outcomes is understandable considering that delirium is associated with long-term cognitive decline, including incident dementia in older adults, and has an adverse impact on health-related quality of life [6]. Similarly, inclusion of “emotional distress” is reasonable as delirium can cause considerable subjective distress such as fear, anxiety, depression, and post-traumatic stress disorder [39]. Emotional distress at the time of delirium may manifest as psychomotor agitation, an outcome that was ranked highly in the Delphi rounds despite being voted out of the final COS. Emotional distress and burden to family members is also substantial [40]; however, researcher participants in our study, in particular, rated this outcome as unimportant for COS inclusion. However, the COS represents the minimum set of outcomes recommended for inclusion in all studies; hence, these outcomes can be included in future studies despite not included in the final COS.

A group with high delirium prevalence is the critically ill [41]. Our group recently completed a COS for future trials of interventions for preventing or treating delirium experienced by critically ill adults [42]. Interestingly, the outcomes selected for this COS, that used the same processes but an expert panel with experience of critical care, were similar, with five outcomes included in both COS. Rather than “delirium duration,” participants in the critical care COS selected the outcome “time to delirium resolution” as this was felt more indicative of the end of delirium. Mortality was included as a seventh outcome which likely reflects the increased risk of death of critically ill patients [43]. Ongoing work for both these COS is to establish the measurement instruments and the time horizon for measurement for each of the outcomes.

Strengths of this study are inclusion of interviews with delirium survivors and family members in the item generation phase, a relatively large and international stakeholder panel, and adherence to COMET COS development methods. Study limitations include exclusion of non-English studies reporting outcomes as well as observational studies and non-English speaking research participants. International representation in the interview phase was limited to three nations. The majority of published studies included in our systematic review phase included post-operative patients which may limit the generalizability of our findings. Our decision to exclude outcomes identified in < 5% of studies with our systematic review may have led to exclusion of important outcomes. However, this was mitigated by their inclusion if mentioned in survivor/family member interviews and enabling Delphi round 1 participants to suggest additional outcomes for consideration. We did not ask Delphi participants to identify themselves as a survivor or family member. As such, we cannot confirm if we have sufficient representation of these distinct groups. Due to the timing of the Delphi and the in-person consensus meeting, we were unable to conduct a third Delphi round confirming importance scores of the eight additional outcomes suggested in round 1. Although patient and family representation in the Delphi was reasonable, only one delirium survivor was able to attend the in-person consensus meeting.

## Conclusion

With development of this COS, we seek to promote standardized outcome selection and reporting in future trials of interventions to prevent and/or treat delirium in adults experiencing an acute care hospital admission and without an ICU admission. We anticipate widespread dissemination and adoption of this COS will facilitate faster detection of effective interventions to prevent or treat delirium due to enhanced ability to pool trial data, ultimately improving patient outcomes. Further work is now needed to operationally define the six core outcomes that will include consensus in selecting validated measurement instruments, the time horizon for measurement, analysis metrics, and method of aggregation.

## Supplementary Information

**Additional file 1: Table 1.** Delirium Specific Outcomes extracted from 183 studies during the item generation stage. **Table 2.** Other outcomes extracted from 183 studies during the item generation stage grouped according to COMET taxonomy. **Table 3.** Deduplication decisions for Delphi Round1 Questionnaire. **Table 4.** Round 1 Delphi Scores.
