# Supplementary material for: A core outcome set for studies evaluating interventions to prevent and/or treat delirium for adults requiring an acute care hospital admission: an international key stakeholder informed consensus study
Source: BMC Med. 2021 Jun 18;19:143. doi: 10.1186/s12916-021-02015-3 (PMC8211534; doi:10.1186/s12916-021-02015-3)
Supplement: Supplementary file 1 — Additional file 1: Table 1. Delirium Specific Outcomes extracted from 183 studies during the item generation stage. Table 2. Other outcomes extracted from 183 studies during the item generation stage grouped according to COMET taxonomy. Table 3. Deduplication decisions for Delphi Round1 Questionnaire. Table 4. Round 1 Delphi Scores. [file 12916_2021_2015_MOESM1_ESM.docx]

**Additional File Table 1 Delirium Specific Outcomes** (N = 183 studies)

| **Outcome** | **n (%)** | **Primary outcome** |
| --- | --- | --- |
| Delirium incidence | 144 (79) | 108 (75) |
| Delirium severity | 60 (33) | 14 (23) |
| Delirium duration | 53 (29) | 11 (21) |
| Delirium prevalence | 20 (11) | 9 (45) |
| Time to delirium onset | 14 (8) | 2 (14) |
| Delirium resolution | 13 (7) | 1 (11) |
| Delirium type | 8 (4) | 1 (11) |
| Use of anticholinergic or other psychoactive/tropic agents | 8 (4) | - |
| Delirium reoccurrence | 4 (2) | - |
| Delirium other^a^ | 3 (2) | - |

a 1 study reported time free from delirium ; 1 study reported family distress associated with delirium; 1 study reported frequency of individual symptoms of delirium including inattention, disorientation, motor activity.

**Additional File Table 2 Other Outcomes grouped according to COMET taxonomy** (N = 183 studies)

| **Outcome** | **n (%)** | **Primary outcome^a^** |  |
| --- | --- | --- | --- |
| 1. **Mortality** | | |  |
| Mortality | 60 (33) | - |  |
| Survival | 7 (4) | - |  |
| **3. Cardiac Outcomes** | | | |
| Hypo/hypertension & use of inotrope/vasopressors | 16 (9) | 1 (6) |  |
| Arrhythmia | 14 (8) | - |  |
| Hemodynamic/gas exchange parameters | 11 (6) | 2 (18) |  |
| **8. Gastrointestinal outcomes** | | | |
| Nausea and vomiting | 20 (11) | - |  |
| **9. General Outcomes** | | |  |
| Surgical recovery | 4 (2) | - |  |
| Frailty | 2 (1) | - |  |
| **10. Hepatobiliary outcomes** | | | |
| Liver function tests | 1 (1) | - |  |
| **11. Immune system outcomes** | | | |
| Biomarkers | 15 (8) | 1 (13) |  |
| **12. Infection Outcomes** | | | |
| Infectious complications | 30 (16) | 1 (3) |  |
| Illness severity | 5 (3) | - |  |
| **13. Injury Outcomes** | | | |
| Falls | 30 (16) | 1 (3) |  |
| **14. Metabolism and nutrition outcomes** | | |  |
| Nutritional status | 3 (2) | - |  |
| **17. Nervous System Outcomes** | | | |
| Sleep quality and quantity | 15 (8) | - |  |
| Circadian rhythm | 4 (2) | 1 (25) |  |
| Coma | 1 (1) | - |  |
| **19. Renal and urinary outcomes** | | | |
| Fluid balance and electrolytes | 15 (8) | - |  |
| Renal failure | 11 (6) | - |  |
| Incontinence/urinary retention | 10 (6) | - |  |
| **21. Psychiatric outcomes** | | |  |
| Antipsychotic drug use and additional (rescue) medication to manage agitation | 27 (15) | - |  |
| Use of anticholinergic medications | 8 (4) | - |  |
| Behavioural disturbance | 3 (2) | - |  |
| Agitation (incidence and duration) | 3 (2) | 1 (33) |  |
| **22. Respiratory outcomes** | | | |
| Pneumonia | 18 (10) | 1 (6) |  |
| Duration of mechanical ventilation | 8 (4) | - |  |
| Desaturation | 5 (3) | - |  |
| Reintubation | 1 (1) | - |  |
| **23. Skin and subcutaneous tissue outcomes** | | | |
| Pressure ulcers | 16 (9) | 1 (6) |  |
| **24. Vascular outcomes** | | | |
| Thrombosis | 9 (5) | - |  |
| Stroke | 9 (5) | - |  |
| **25. Physical Functioning** | | | |
| Activities of daily living | 42 (23) | 7 (17) |  |
| Functional milestones | 8 (4) | - |  |
| Walking distance | 7 (4) | 1 (14) |  |
| Mobilization | 5 (3) | - |  |
| Muscle strength | 3 (2) | - |  |
| **28. Emotional Functioning/Wellbeing** | | | |
| Depression | 12 (7) | - |  |
| Anxiety | 6 (3) | - |  |
| Wellbeing/general stress | 2 (1) | - |  |
| PTSD | 1 (1) |  |  |
| **29. Cognitive Functioning** | | | |
| Cognitive status | 43 (24) | 7 (16) |  |
| Post-operative cognitive dysfunction | 19 (10) | 9 (47) |  |
| Incident dementia | 1 (1) | - |  |
| **30. Global Quality of life** | | | |
| Health-related quality of life | 15 (8) | - |  |
| **32. Delivery of Care Outcomes** | | | |
| *Pain related* |  |  |  |
| Pain | 37 (20) | 5 (14) |  |
| Use of analgesia/rescue analgesia | 18 (10) | - |  |
| Dose of analgesia | 20 (11) | 1 (5) |  |
| *Sedation related* |  |  |  |
| Sedation incidence/level/score | 19 (10) | 1 (5) |  |
| Physical restraint (use and duration) | 16 (9) | 1 (6) |  |
| Sedative dose | 3 (2) | - |  |
| Sleep medications | 2 (1) | - |  |
| Treatment interference | 2 (1) | - |  |
| Use of neuromuscular blockade | 1 (1) | - |  |
| *Surgery and anaesthesia* |  |  |  |
| Duration of surgery | 18 (10) | - |  |
| Duration of anesthesia | 15 (8) | - |  |
| Anesthesia recovery/emergence (quality of and time to) | 14 (8) | 1 (7) |  |
| Anesthetic dose/type | 13 (7) | - |  |
| Need for anesthesia | 4 (2) | - |  |
| *Delivery of care-other* |  |  |  |
| Protocol/drug adherence and other process outcomes | 59 (32) | 3 (5) |  |
| Satisfaction | 16 (9) | 1 (6) |  |
| Number of ward moves | 2 (1) | - |  |
| Nursing workload | 1 (1) | - |  |
| **34. Resource Use - Costs** | | | |
| Cost | 17 (9) | - |  |
| **35. Resource Use –Hospital** | | | |
| Length of stay (hospital, ICU, PACU, rehab) | 133 (73) | 7 (5) |  |
| Discharge disposition | 40 (24) | 1 (2) |  |
| Hospital readmission | 26 (14) | - |  |
| Need for ICU admission | 9 (5) | - |  |
| In-patient resource utilization | 2 (1) | - |  |
| **37. Societal/carer burden** | | | |
| Use of home care/social support services | 6 (3) | 1 (17) |  |
| Caregiver outcomes | 4 (2) | - |  |
| Use of primary care resources and unmet social needs | 2 (1) | - |  |
| **38. Adverse Events/effects** | | | |
| Surgical complications including blood loss and need for transfusion | 46 (25) | - |  |
| Non-specified adverse events | 43 (24) |  |  |
| Cardiac adverse events | 26 (14) | 3 (12) |  |
| Other adverse events | 26 (14) |  |  |
| Respiratory adverse events | 21 (11) | - |  |
| Gastrointestinal adverse events | 14 (8) | - |  |
| Renal and urinary adverse events | 8 (4) | 1 (13) |  |
| Cerebrovascular adverse events | 7 (4) | 1 (14) |  |
| Extrapyramidal side effects | 6 (3) | - |  |
| Geriatric syndrome -composite of delirium functional decline, in-hospital falls, in-hospital pressure injury and new incontinence | 1 (1) | 1 (100) |  |

a Denoted as the primary outcome of studies that reported this outcome

**Additional File Table 3 Deduplication decisions for Delphi Round1 Questionnaire**

| **Outcome** | **Source** | **Item reduction** | **Reason for redundancy** | **Final R1 Wording** |
| --- | --- | --- | --- | --- |
| Delirium incidence | SR | INC-MOD |  | Delirium occurrence |
| Delirium prevalence | SR | REDUN | Due to variable use of terms prevalence & incidence combined as delirium occurrence |  |
| Delirium severity | SR | INC |  | Delirium severity |
| Delirium duration | SR | INC |  | Delirium duration |
| Time to delirium onset | SR | INC |  | Time to delirium onset |
| Delirium resolution | SR | INC |  | Delirium resolution |
| Delirium type | SR | INC |  | Delirium type |
| Delirium reoccurrence | SR | INC |  | Reoccurrence of delirium |
| Mortality | SR | INC |  | Mortality |
| Survival | SR | REDUN | Considered to overlap with mortality |  |
| Hypo/hypertension & use of vasopressors/inotropes | SR | COMPL |  |  |
| Arrhythmia | SR | COMPL |  |  |
| Hemodynamic/gas exchange parameters | SR | COMPL |  |  |
| Gas exchange parameters | SR | MONIT |  |  |
| Nausea and vomiting | SR | COMPL |  |  |
| Liver function tests | SR | MONIT |  |  |
| Biomarkers & blood chemistry | SR | MONIT |  |  |
| Infectious complications | SR | COMPL |  |  |
| Organ failure (illness severity) | SR | COMPL |  |  |
| Falls | SR | INC-MOD |  | Falls and other injuries |
| Nutritional status | SR | RISK |  |  |
| Sleep quality and quantity | SR | INC |  | Sleep |
| Circadian rhythm | SR | <5% |  |  |
| Coma | SR | <5% |  |  |
| Stroke | SR | COMPL |  |  |
| Fluid balance & electrolytes | SR | MONIT |  |  |
| Renal failure | SR | COMPL |  |  |
| Incontinence/urinary retention | SR | COMPL |  |  |
| Antipsychotic drug use and additional (rescue) medication to manage agitation | SR | INC |  | Use of antipsychotics or other medications for agitation |
| Use of anticholinergic medications | SR | RISK |  |  |
| Behavioural disturbance | SR | <5% |  |  |
| Agitation occurrence | SR <5% BUT Interview | INC |  | Agitation occurrence |
| Pneumonia | SR | INC |  | Pneumonia |
| Ventilation duration | SR | <5% |  |  |
| Desaturation | SR | COMPL |  |  |
| Reintubation | SR | <5% |  |  |
| Pressure ulcers | SR | INC |  | Pressure ulcers |
| Thrombosis | SR | COMPL |  |  |
| Stroke | SR | COMPL |  |  |
| Activities of daily living | SR | INC |  | Activities of daily living |
| Functional milestones | SR | INC-MOD |  | Physical functioning |
| Walking distance | SR | REDUN | Considered covered as physical functioning |  |
| Mobilization | SR | REDUN | Considered covered as physical functioning |  |
| Muscle strength | SR | REDUN | Considered covered as physical functioning |  |
| Depression | SR | INC-MOD |  | Emotional wellbeing of patients including anxiety, depression, acute stress, PTSD |
| Anxiety | SR | REDUN | Considered acceptable to combine measures of emotional wellbeing into one outcome |  |
| Post-traumatic stress | SR | REDUN | Considered acceptable to combine measures of emotional wellbeing into one outcome |  |
| Well-being/general stress | SR | REDUN | Considered acceptable to combine measures of emotional wellbeing into one outcome |  |
| Cognitive status | SR | INC-MOD |  | Cognitive status including memory |
| Post-operative cognitive status | SR | REDUN | Considered acceptable to combine with cognition status |  |
| Incident dementia | SR | REDUN | Considered acceptable to combine with cognition status |  |
| Health-related quality of life | SR | INC |  | Health related quality of life |
| Pain presence | SR | INC-MOD |  | Pains score indicating quality of analgesia |
| Use of analgesia | SR | INC |  | Analgesic drug use |
| Dose analgesia | SR | REDUN | Considered included with analgesic drug use |  |
| Sedation incidence/level/score | SR | INC-MOD |  | Sedation-agitation score indicating quality of sedation |
| Physical restraint | SR | INC |  | Use of physical restraint |
| Sedation dose | SR | REDUN | Considered included with quality of sedation |  |
| Use of sleep medication | SR | <5% |  |  |
| Treatment interference (lines, catheters removal) | SR | REDUN | Considered included as ‘other injuries’ with falls |  |
| Neuromuscular blockade | SR | <5% |  |  |
| Duration of surgery | SR | RISK |  |  |
| Duration of anaesthesia | SR | RISK |  |  |
| Anaesthesia recovery/emergence | SR |  |  |  |
| Anaesthetic dose/type | SR | RISK |  |  |
| Need for anaesthesia | SR | RISK |  |  |
| Protocol/drug-adherence and other process outcomes | SR | INC-MOD |  | Study intervention related process outcomes |
| Satisfaction | SR | INC |  |  |
| Number of ward moves | SR | RISK |  |  |
| Nurse workload | SR | <5% |  |  |
| Costs | SR | INC |  | Costs |
| Length of stay | SR | INC |  | Length of stay |
| Discharge disposition | SR | INC |  | Hospital disposition |
| Readmission | SR | INC |  | Hospital readmission |
| Need for ICU admission | SR | <5% |  |  |
| In-patient resource utilization | SR | <5% |  |  |
| Use of home care/social support services | SR | <5% |  |  |
| Caregiver outcomes | SR | REDUN | Considered included un emotional well-being of family members |  |
| Use of primary care resources and unmet social needs | SR | <5% |  |  |
| Serious adverse events/side effects | SR | INC-MOD |  | Adverse events/side effects of an intervention to prevent or treat delirium |
| Surgical complications including blood loss and need for transfusion | SR | COMPL |  |  |
| Cardiac adverse events | SR | COMPL |  |  |
| Respiratory adverse events | SR | COMPL |  |  |
| Gastrointestinal adverse events | SR | COMPL |  |  |
| Renal & urinary adverse events | SR | COMPL |  |  |
| Cerebrovascular adverse events | SR | COMPL |  |  |
| Extrapyramidal effects | SR | COMPL |  |  |
| Emotional wellbeing of family members | Interview | INC |  | Emotional well-being of family members |
| Able to return home/live independently | Interview | REDUN | Considered redundant with activities of daily living/physical functioning |  |
| Time to recognition of delirium | Interview | REDUN | Considered redundant with time to delirium onset |  |
| Repeated infection as a delirium risk factor | Interview | RISK |  |  |

INC-MOD = included but wording modified; REDUN = redundant/overlapping outcome; INC = include as it; <5% = identified in <5% of studies and not mentioned in interview; COMPL = specific complication, side effect or adverse event; MONIT = monitoring type outcome; AGGR = aggregate study population outcome as opposed to individual patient outcome. RISK = risk factor or exposure for delirium as opposed to study outcome

**Additional File Table 4 Round 1 Delphi Scores**

|  | Overall | | Survivor/Family  (N=8) | Clinician  (N=67) | Researcher  (N=35) |
| --- | --- | --- | --- | --- | --- |
| Outcomes | Mean (SD) | % Critical | % Critical | % Critical | % Critical |
| Delirium occurrence | 8.5 (0.8) | 95 | 88 | 95 | 97 |
| Mortality | 8.2 (1.1) | 92 | 100 | 91 | 94 |
| Cognitive status | 8.0 (1.1) | 88 | 75 | 86 | 97 |
| Delirium duration | 8.0 (1.1) | 86 | 75 | 86 | 88 |
| Adverse events/side effects | 7.8 (1.3) | 85 | 75 | 86 | 85 |
| Delirium severity | 7.8 (1.3) | 83 | 88 | 83 | 82 |
| Activities of daily living | 7.7 (1.3) | 82 | 63 | 84 | 85 |
| Falls and other injuries | 7.6 (1.4) | 80 | 75 | 81 | 79 |
| Use of antipsychotics/other medication for agitation | 7.6 (1.5) | 80 | 88 | 86 | 66 |
| Hospital disposition | 7.6 (1.3) | 78 | 57 | 82 | 75 |
| Patient emotional wellbeing | 7.5 (1.3) | 78 | 100 | 83 | 63 |
| Delirium resolution | 7.6 (1.4) | 77 | 63 | 82 | 73 |
| Length of stay | 7.5 (1.4) | 77 | 88 | 78 | 72 |
| Agitation | 7.3 (1.6) | 75 | 100 | 80 | 59 |
| Physical functioning | 7.4 (1.3) | 74 | 63 | 78 | 69 |
| Health-related quality of life | 7.3 (1.2) | 72 | 100 | 78 | 53 |
| Sleep | 7.2 (1.6) | 72 | 100 | 75 | 59 |
| Physical restraint | 7.2 (1.8) | 71 | 71 | 78 | 59 |
| Hospital readmission | 7.3 (1.5) | 71 | 50 | 73 | 72 |
| Sedation score/level indicating quality of sedation | 7.0 (1.8) | 70 | 75 | 75 | 58 |
| Delirium reoccurrence | 7.3 (1.6) | 68 | 75 | 71 | 64 |
| Costs | 7.0 (1.5) | 60 | 50 | 61 | 58 |
| Delirium type | 6.8 (1.8) | 60 | 63 | 65 | 48 |
| Pain score/level indicating quality of analgesia | 6.7 (1.7) | 57 | 75 | 61 | 44 |
| Pressure ulcers | 6.7 (1.4) | 57 | 63 | 58 | 55 |
| Analgesic drug use | 6.6 (1.7) | 54 | 63 | 59 | 39 |
| Pneumonia | 6.7 (1.5) | 53 | 63 | 56 | 45 |
| Satisfaction | 6.5 (1.7) | 49 | 75 | 58 | 24 |
| Time to delirium onset | 6.6 (1.7) | 49 | 75 | 56 | 30 |
| Family emotional wellbeing | 6.4 (1.4) | 46 | 63 | 56 | 25 |
| Study intervention related process outcomes | 6.4 (1.8) | 46 | 57 | 54 | 29 |

NB: participants self-selected the stakeholder group they most identified with during Round 1
